# Experimental verification of high energy laser-generated impulse for remote laser control of space debris

**DOI:** 10.1038/s41598-018-26336-1

**Published:** 2018-05-31

**Authors:** Raoul-Amadeus Lorbeer, Michael Zwilich, Miroslav Zabic, Stefan Scharring, Lukas Eisert, Jascha Wilken, Dennis Schumacher, Markus Roth, Hans-Albert Eckel

**Affiliations:** 1 German Aerospace Center - Deutsches Zentrum für Luft- und Raumfahrt e.V. (DLR), Institut für Technische Physik, Studien und Konzepte, Pfaffenwaldring 38-40, 70569 Stuttgart, Germany; 2 GSI Helmholtzzentrum für Schwerionenforschung GmbH, Atomic, Plasma Physics and Application, Planckstraße 1, 64291 Darmstadt, Germany; 3 Technische Universitt Darmstadt, Institut für Kernphysik, Schlossgartenstraße 9, 64289 Darmstadt, Germany

## Abstract

Walking along a beach one may notice debris being washed ashore from the vast oceans. Then, turning your head up at night you even might noticed a shooting star or a bright spot passing by. Chances are, that you witnessed space debris, endangering future space flight in lower earth orbit. If it was possible to turn cm-sized debris into shooting stars the problem might be averted. Unfortunately, these fragments counting in the 100 thousands are not controllable. To possibly regain control we demonstrate how to exert forces on a free falling debris object from a distance by ablating material with a high energy ns-laser-system. Thrust effects did scale as expected from simulations and led to speed gains above 0.3 m/s per laser pulse in an evacuated micro-gravity environment.

## Introduction

If we had to make a slogan for our work it might be: “Space is precious.” In our case of - outer - space this is a rather abstract statement. To convey a picture of the valuable infrastructure “lower Earth orbit” (LEO) a hypothetical question might help: “How would your life be different without satellites?” This again leads us to the problem we want to address: Debris in lower Earth orbit.

It poses a serious threat to the growth of future space programs. The international space station (ISS) already has to perform several maneuvers per year to dodge debris gradually descending down to Earth^1^. The Iridium Cosmos collision^2^ showed, that with current tracking capabilities even the choice whether to dodge or not might fail and, not short of losing the spacecraft, creates a devastating amount of smaller new fragments^3^. Even minor fragments with several mm in diameter can destroy a satellite’s functionality^4,5^ or lead to a catastrophic breakup of small multi kg satellites or other debris fragments^6^. Since small fragments with diameters of several centimeters or less cannot be tracked with current technologies it is difficult up to impossible to determine whether a satellite’s malfunctioning is actually caused by small space debris or by internal faults^5,7^. Statistical models point to the endangered regions in orbits between the edge of space at a few hundred kilometers up to roughly 1500 km above the Earth’s surface^8,9^. This region hosts current human space flight, the Hubble space telescope^10^, weather satellites, global communication satellites such as IRIDIUM^2,11^ or in the future, OneWeb and other constellations^12^, as well as scientific satellite missions, helping us to understand, e.g, our ecosystem or the ongoing climate change^13^. The high density of space debris in LEO orbits is no coincidence, since most of the debris is caused by the human activities.

Many methods to remove space debris from LEO have been proposed over the past decades. Some involve the active collection of bigger debris parts to avoid their fragmentation^14,15^, some shall use artificial drag to lower the objects orbit and eventually remove them from Earth orbit by atmospheric reentry^16,17^. A third possibility using a laser to exert thrust forces by laser ablation was proposed several decades ago by Schall and Phipps *et al*.^18,19^. The uniqueness of the laser-based debris removal (LDR) concept lies in the possibility to operate a ground-based facility and the achievement of high removal rates in the range of hundreds of particles per day^20^. Such a system allows to remove a high quantity of small debris estimated in the 100 thousands of particles^14,21^.

If the debris problem will not be accounted for, extrapolations show that fragmentation will increase the debris amount far beyond our century even with no additional material launched^3^. This could eventually lead to the loss of high quality weather forecast, navigation systems, earth observation, satellite TV, space science etc. This in turn would have a significant negative impact on the lives of the generations to come and, therefore, we are responsible to cope with this undesirable prediction. Being at the edge of debris runaway effects, we chose to perform experiments reproducing a realistic situation as closely as possible in order to evaluate the usability of LDR.

The underlying process which leads to the laser-ablative generation of thrust is well understood and has been investigated for decades now^22,23^. An intense laser pulse super-heats the surface of a target, which leads to the vaporization of a very thin surface layer. Due to the expansion of the hot material it is accelerated. According to the third Newtonian law (actio = reactio) a force is exerted onto the target. This processes is a common side-effect in laser material processing as, e.g., during car manufacturing. Nevertheless, most laser ablative thrust experiments had restrictions concerning pulse energy, object size, shape or movement^18,24–27^. Furthermore, many assumptions were necessary to evaluate the controllability of the process. Our intention is to demonstrate the feasibility of the impulse generation process with realistic constraints. Therefore, size, material, shape, fluence, mechanical restrictions and surrounding pressure were chosen to reproduce the circumstances of a space debris fragment. In addition to the general proof of feasibility, the dependency of generated impulse was tested against further parameters such as the variation of material, pressure, and angle of incidence.

In a previous publication^28^, we presented a numerical method to simulate the induced impulse transfer for arbitrarily shaped objects by extrapolating the currently known laser matter interaction parameters. These simulations build the basis for our experiment and were used to interpret our results.

## Results

### Findings from the experiments

**Figure 1:**
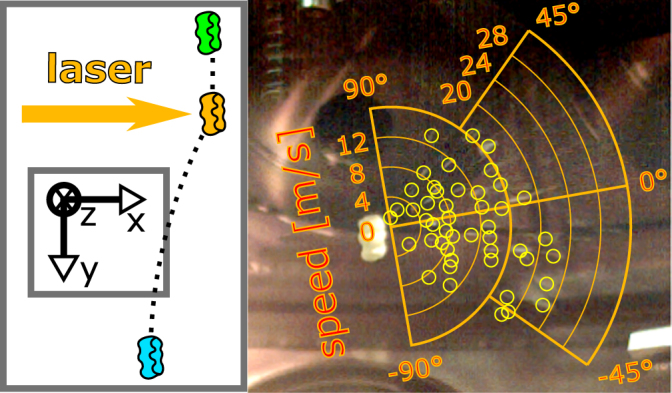
Fragmentation of green gummy bear. Direction of laser irradiation is indicated by the orange arrow. A radial scale (orange) indicates the projected velocities in viewing direction. Manually identified fragments are highlighted with yellow circles.

**Figure 2:**
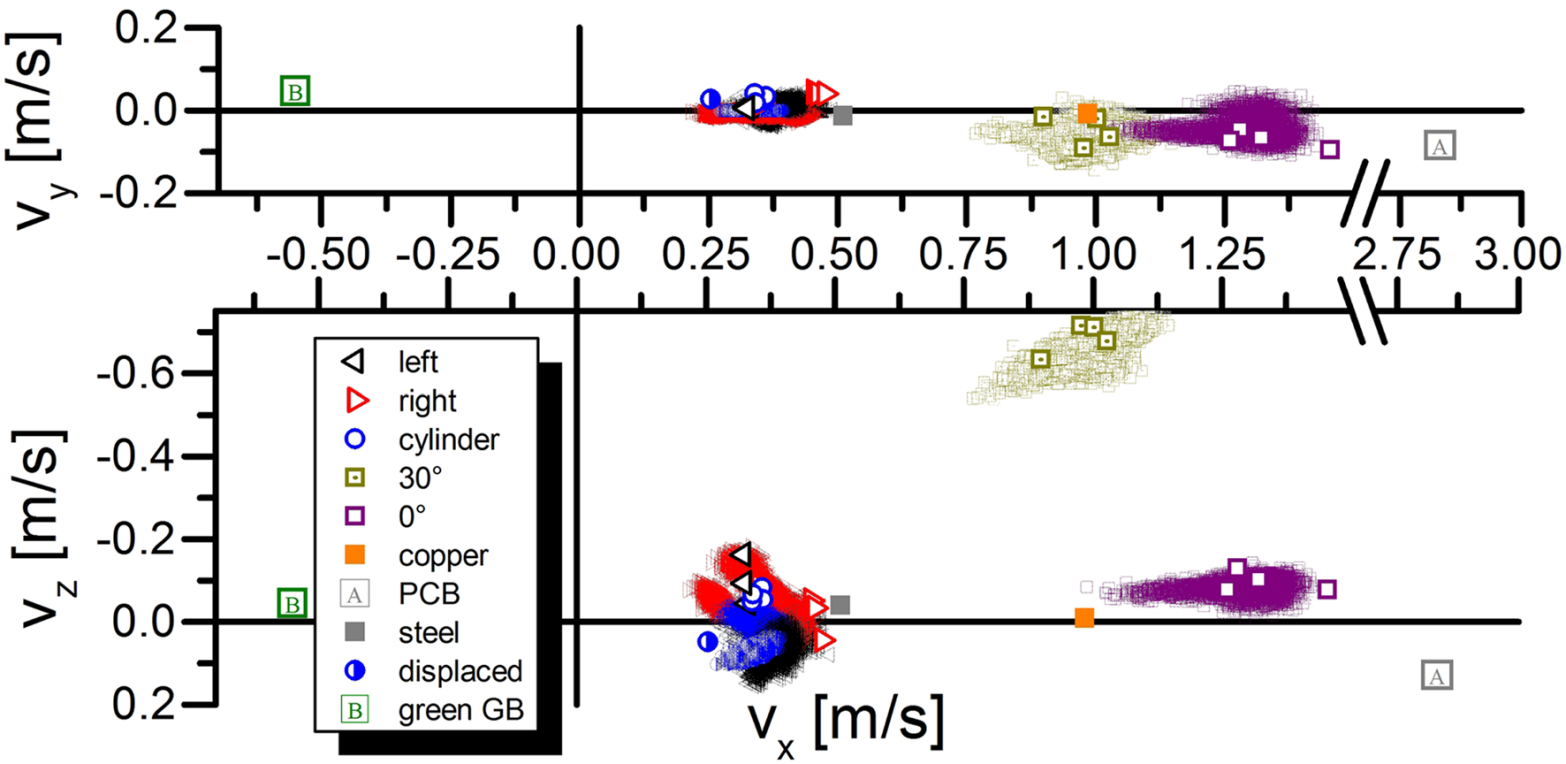
Object velocity changes after laser irradiation. Simulation results are indicated as point clouds.

**Figure 3:**
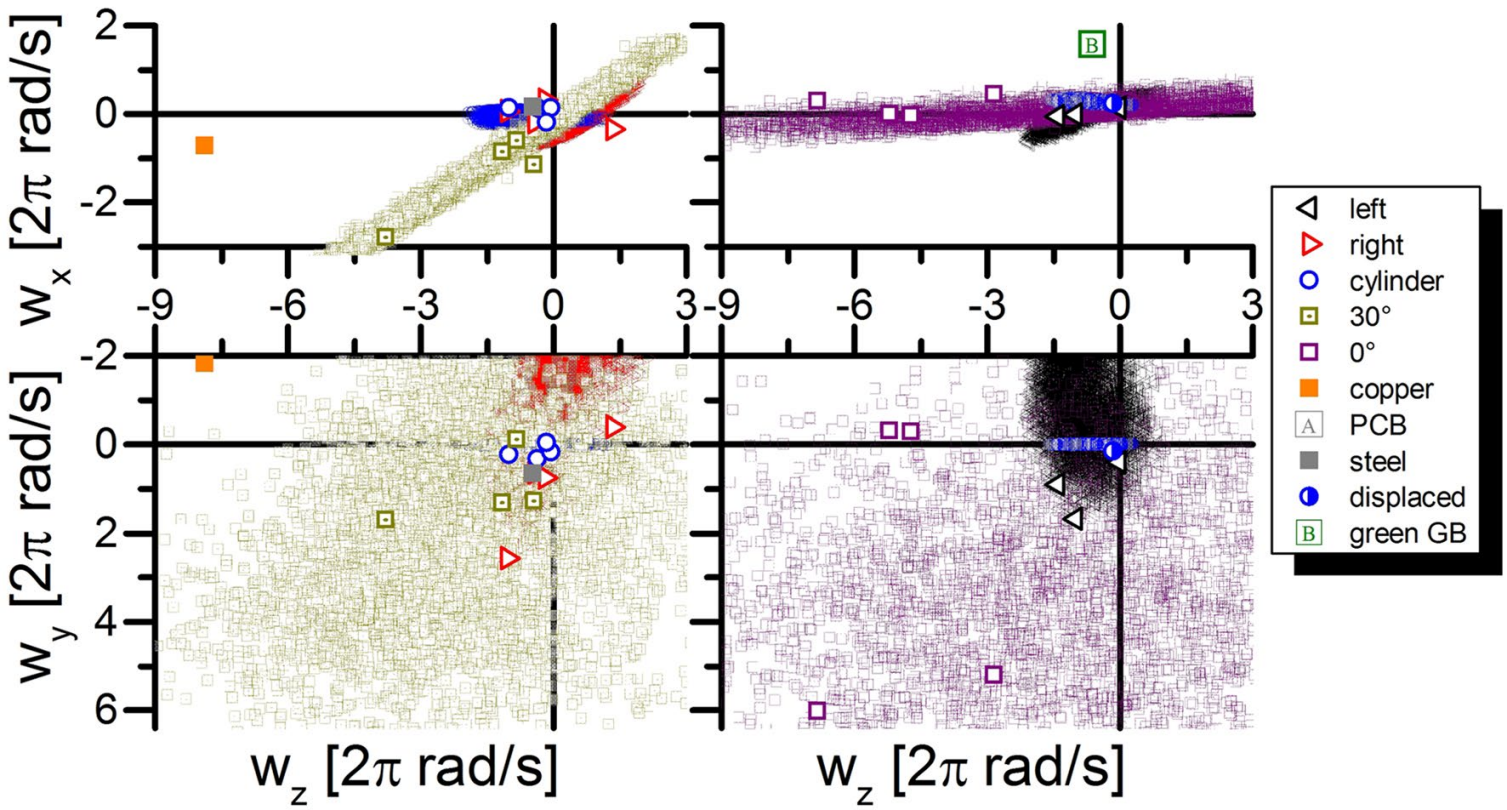
Change in angular velocity $\Delta \overrightarrow{\omega }$ after laser irradiation. Simulation results are indicated as point clouds.

To ensure micro-gravity preparation of the target it was dropped from half a meter of height and, after approximately 150 ms of free fall, illuminated by an intense laser-pulse as displayed in Fig. 1 on the left (more details see materials and methods). Kinetic effects were evaluated by stereoscopic imaging and are displayed in Figs 2 and 3. Measured values are also summarized in Supplementary Table S1.

First of all we were able to identify that all 27 tests led to the generation of impulse. All targets were deflected from their original direction of movement strongly enough to be observed directly in the slow motion videos from the high-speed cameras (compare supplementary movie S1). The amount of impulse or speed changed dominantly with the targets material and weight. One material change actually led to the reversal of the typical (observed in all other tests) direction of movement (compare Fig. 2).

Another aspect which appears in the videos, lies in the pictures generated from the ablation process itself. The extremely bright plasma is evident in the high-speed videos, and shows slight variations depending on the initial parameters.

The first parameter showing significant differences is the surrounding pressure. For pressures below 3·10^−3^ mbar the plasma of the ablation process disappears within one frame or 1 ms, whereas at between 1·10^−1^ mbar and 2·10^−1^ mbar the plasma is visible over the duration of up to two frames. With a visible expansion trace of approximately 0.3 m this leads to an estimate in the order of several 100 *m*/s (0.3 *m*/0.001*s* = 300*m*/s). Nevertheless, the measured velocities of the targets did show statistical deviations below 7% within their groups including experiments at both pressure levels (4 samples each, ${\sigma }_{v}^{0^\circ }=0.044\,m/s$, ${\sigma }_{v}^{30^\circ }=0.088\,m/s$).

The second parameter is the variation of the material. Since the material variation has been performed only once per material we will focus on the most evident change in the ablation processes. Experiment #27 was performed with a transparent bio-polymer typically referred to as (green) gummy bear. During ablation one can identify high intensities saturating the camera picture at the front and the back, relative to the laser irradiation, of the target. Contradictory to all preceding experiments small fragments are emitted. Furthermore, these fragments are ejected from the backside facing away from the laser beam, leading to an impulse of the target directed towards the laser. A graphical analysis (compare Fig. 1) results in well above 50 fragments traveling with speeds ranging from 1 to 25 m/s. With an average speed of 12.5 m/s and the assumption that all impulse was generated by these particles, we may conclude, that the average mass per particle is ${\bar{m}}_{p}=\frac{0.6\,m\,/\,s}{12.5\,m\,/\,s}\cdot \frac{2.19\,g}{50}=2.1\,mg$. With a density of $1.3\,{\rm{to}}\,{\rm{1.6}}\,\frac{mg}{m{m}^{3}}$^29^ this is in agreement to the video observation of particles with a size below 1 mm.

The measurement of the mass differences before and after the laser ablation for the aluminum plates was Δ*m* = 91 μg ± 25 μg. The kinetic evaluations result in an average ${\rm{\Delta }}p=1.38\cdot {10}^{-3}\,kgm/s\pm 0.10\cdot {10}^{-3}\,kgm/s$. By this the kinetic jet energy of ${E}_{Jet}=\frac{{\rm{\Delta }}{p}^{2}}{2{\rm{\Delta }}m}=\frac{{(1.38\cdot {10}^{-3}kgm/s)}^{2}}{2\cdot 91\,\mu {\rm{g}}}=10.46J\pm 3.25J$ can be calculated. This reassembles approximately 1/8 of the invested laser energy.

The error of dv and dw from the measurements was calculated using the remaining positions error in the fitted moving frame (compare materials and methods). The resulting position errors (dx, dy, dz) are set into context to the time interval they correspond to before and after the laser pulse is applied. This results in typical velocity errors below 1.2·10^−2^ *m*/*s*. A similar approach can be used to identify the error of rotation. Here, additionally the average distance to the center of rotation was estimated to be 10^−2^ *m*. In this case typical rotation errors are below *π*/2^*rad*^/*s*. Only two experiments showed higher tolerances namely shots #15 and #24, these yield errors below 5·10^−2^ *m*/*s* and 2*π*^*rad*^/*s*. This does not account for systematic errors as, e.g., calibration errors of the stereoscopic setup inducing fictitious forces due to data processing.

The tracking of the targets allows to deduce several parameters of interest. The output parameters from the rigid body fitting procedure are the laser-induced velocity and angular velocity. Fitted values are listed in Supplementary Table S1 and plotted in Figs 2 and 3.

At first sight lighter objects show higher velocities and the dominant direction of acceleration is along the axis of laser radiation (compare Fig. 3), which coincides with the x-axis of our coordinate system. Repetitions of the same experiment are represented in identical colors. In this case one can deduce several observations. Shifting the center of a rotation symmetric object as, e.g., a cylinder leads to a change of thrust direction. A similar observation can be made by comparing the aluminum plates at the normal (0°) and tilted (30°) angle.

The velocity of the objects is, as expected, strongly influenced by their weight. Nevertheless, the material has a strong influence, too. Copper does not yield as high of an impulse as the aluminum plates which again led to less impulse compared to the steel plate only surpassed (note the interruption of the *v*_*x*_-axis in Fig. 2) by the PCB board. This indeed, corresponds to the relations as determined in experiments by D’Souza^30^. Nevertheless, the most surprising result was the switching of the overall direction in impulse for the green gummy-bear.

A third aspect influencing the amount of generated impulse is the change of the L corner targets orientation. The experiments show a significant raise of impulse for the L corners with the corner facing away from the incoming laser light. L-corners facing towards the laser beam have a velocity of 0.336 *m*/*s* ± 0.008 *m*/*s* (n = 3) the L-corners facing away from the laser beam show an average velocity of 0.434 *m*/*s* ± 0.040 *m*/*s* (n = 4). In the latter case the uncertainty is dominated by shot #7 which was performed with 75% laser energy compared to the remaining group. Therefore, we conclude a statistical significant difference in impulse generation for these two cases.

The rotations induced by the process are displayed in Fig. 3. Here, the different experiments are not as separable as they are by their velocities (Fig. 2). Despite this fact, multiple effects can be observed. First of all, all heavier (>3 g) targets showed only minor rotation rates. The lighter copper and aluminum plates were subject to much higher rotation rates. Here again one can separate between the PCB-board (not shown compare Supplementary Table S1), the tilted plates and all other experiments. Typically, there is no rotation around the x-axis coinciding with the laser beam. Therefore, the preparation of the tilted plates appears to allow a stronger rotation around the axis of the laser beam propagation.

### Findings from the simulations

Simulations for the targets showed an underestimation of the total impulse generated. Therefore, we chose to adjust the fitting parameter *b* as deduced from Polly-2T^31^ simulations in Supplementary Table S3 by a factor of 4/3 for our EXPEDIT simulations, which led to the results indicated as point clouds in Figs 2 and 3 (compare materials and methods).

Additionally, the rotation of the targets is part of the simulation, too. Figure 3 shows the distribution of all possible simulation results considering our uncertainties in a priory conditions. Clearly, the results for the rotation vector are far more sensitive to these conditions than the overall momentum.

A comparison between the velocity vectors and the orientations of the flat targets show the expected correlation to the initial orientation. In contrast to this the simulation of the L-corners occupy a large variety of directions, which is likely caused by the uncertainties of the laser hit position combined with the discontinuous orientation at the corner tips. A slight deviation leads to a significant change of average momentum direction, which might explain the discrepancy between simulation and experiment of $\Delta \overrightarrow{v}$ for L-corners facing towards the incoming laser-light. This is similar to the purposefully displaced cylinder (#26), which did not react as sensitive to small displacements. Additionally, the simulations do not show a distinct separation in momentum amplitude between the corners facing towards and away from the laser beam.

The simulated angular frequencies in Fig. 3 show high uncertainties compared to the experimental data. This is most likely due to the uncertainty of the exact distribution and position of the laser fluence on the target. Slight variations in the order of 1 mm lead to a large variety of possible solutions. Nevertheless, global tendencies can be found. The simulations show, that the possible solutions are dominantly distributed within two dimensional or in the case of cylinders one dimensional solution spaces. In nearly all cases these fit quite nicely, nevertheless the L-corners facing away from the laser source again show a systematic discrepancy from the simulated results.

Moreover, the objects with tilted surfaces as the aluminum plates tilted by 30° and the L-corner targets show solution spaces tilted away from the $({\omega }_{y},\,{\omega }_{z})$ -plane.

## Discussion

We now will discuss our findings in the context of our most eminent question in mind: Is LDR based on laser ablation feasible? This question obviously implies several other questions, of greater technical detail.

Would a LDR system generate enough thrust to remove a significant amount of space debris? The conditions tested led to the generation of thrust in all cases. Therefore, we are quite confident that not only one single type but rather a large variety of space debris materials can be addressed by this method. We were able to show velocity increments of Δ*v* ≧ 0.3 *m*/*s* for particles between 1.1 g and 3.4 g in mass and one to four square centimeters of laser ablative surface. Objects of sizes between 1 to 5 cm are very dangerous for space assets^32^ and estimated to surpass 600,000 particles in orbit at the moment^14^. Other models already demonstrated that typical Δ*v* of 240 *m*/*s*^33^ or in our case less than 800 laser shots would lead to reentry and burn up of the debris particles. This allows for a simple extrapolation to a total of roughly 1 billion laser shots to vastly reduce the represented class of space debris. At 50 shots per second and a system duty cycle of 15% this procedure would take approximately two years and therefore could be performed within realistic time margins. Experiments introducing two or even multiple succeeding pulses could show if this extrapolation is valid for repetitive ablation as well.

**Figure 4:**
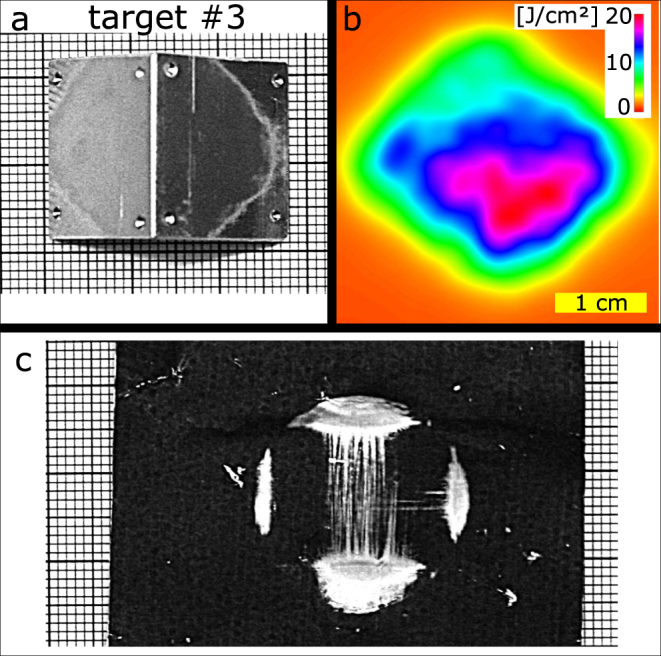
Exemplary data from target #3. (**a**) Target after ablation. Contrast was enhanced to emphasize the ablation border. (**b**) Corresponding fluence map as deduced from beam profiling camera. (**c**) Burn pattern with target obscuring laser beam.

Will it be possible to reach usable irradiation conditions? The photographs of the targets (compare Fig. 4a) show that in the outer regions no ablation took place. The fluence at the laser spot rim did not exceed the ablation threshold. This illustrates that the fluence at the target posts an essential working parameter for such a system. Here, possibly the most eminent problem will be introduced by Earth’s atmosphere, which led to proposals of space based laser systems^23,33–35^ to begin with. The success of a ground-based solution will be strictly connected to the available solutions for atmospheric propagation^36^ which should be addressed in future work. Despite the challenge of fluence delivery, a ground-based solution has fewer limitations concerning electrical power, radiation resistance and system lifetime as well as system maintenance^32^, which need to be considered as well.

How close is the simulation to describing real LDR? The difference for the L-corners facing towards and away from the laser beam is significant and at the moment we are planning to account for this effect. Possible explanations include multiple reflections of the beam as well as nozzle effects. To identify the dominant source further simulations will be necessary. Despite this discrepancy we did indeed reach a high quality simulation of the impulses generated. The generated rotation errors are most likely induced by the remaining alignment error of the targets relative to the laser-beam. Other effects as, e.g., thermal properties were not considered either and are planned to be analyzed in succeeding experiments. A first theoretical study was published recently^37^.

Will the products of the LDR process be harmful to other space assets? Frankly speaking, prior to these experiments we would have neglected this question since the process was expected to only generate plasma, which eventually would cool down to a molecular metal cloud^38^. Our worst case scenario would have been a strangely shaped fragment split into a few pieces before one would be able to react^18^. The gummy bear, representing potentially transparent debris as, e.g., silicon, glass or plastic materials, did not only surprise by its momentum direction opposed to all preceding experiments it also did generate a large amount of very small, likely sub-mm sized, fragments. This kind of fragmentation would be difficult to detect at best. The new question hence should sound: How small do the products of LDR have to be to solve the problem rather than to increase it. Luckily, a recent study indicates that sub-mm sized particles should only lead to surface erosion^32^. Furthermore, small particles are known to have a reduced ballistic coefficient reducing the lifetime of the particle’s orbit significantly^16^.

How was it possible to generate rotations around the direction of the laser beam? At first one does not expect rotation around the direction of the incoming laser light. The dominating amount of experiments confirmed this expectation. Nevertheless, the 30° tilted aluminum plates made it obvious that this is not entirely true. As the direction of impulse is perpendicular to the targets surface only rotations around the surfaces normal are prohibited. This behavior can be reproduced using the EXPEDIT simulation tool. Despite the 30° case the simulations indicated, that the induced rotation is always arranged within a one- or two-dimensional margin of rotation vectors. The margins shape and size is determined by the orientation and shape of the target. Rotational symmetric targets as, e.g., the cylinders tend to induce negligible angular velocities around their axis of symmetry.

## Conclusions

Our next steps will be directed at different materials, fragmentation, simulating the controllability of the deflected orbits and the approaches to optimize laser fluences in LEO altitudes. Therefore, we plan to introduce what we call “passive three beam interference”. This method should allow to increase the fluence at the target at least by a factor of three and shall be described in greater detail in our succeeding work.

In summary, we were able to demonstrate the functionality of laser-ablative impulse generation for multiple representative targets of one to two centimeters in size under realistic conditions. We used an 80 J 1064 nm laser pulse to generate average fluences up to 10 *J*/*cm*^2^. Previous publications indicate that the generation of fluence in the order of 10 *J*/*cm*^2^ might even be possible over distances up to 1000 km^32^. This finally would allow a laser debris removal system to remove vast amounts of cm-sized space debris within a few years.

## Methods

### Experiment

The experiments were performed with the NHELIX laser system at the GSI near Darmstadt Germany^39^.

The NHELIX laser system consists of a ns pulsed laser head generating 10 seed pulses per second. These pulses have an approximate duration of 10 ns and a center wavelength at 1064 nm. A pockels cell is used to introduce temporal shaping of the pulse and reduce the seed rate to 5 Hz. Then a mechanical shutter picks one single pulse out of this pulse train for full energy amplification.

The amplification is achieved by five Nd:glas amplifiers with ascending diameters of which the third one is set up to perform a double-pass arrangement. The aperture of the last amplifying rod is 64 mm which limits the diameter of the final laser beam. The amplified laser-pulse of approximately 80 J pulse energy then is directed into the experiment room.

**Figure 5:**
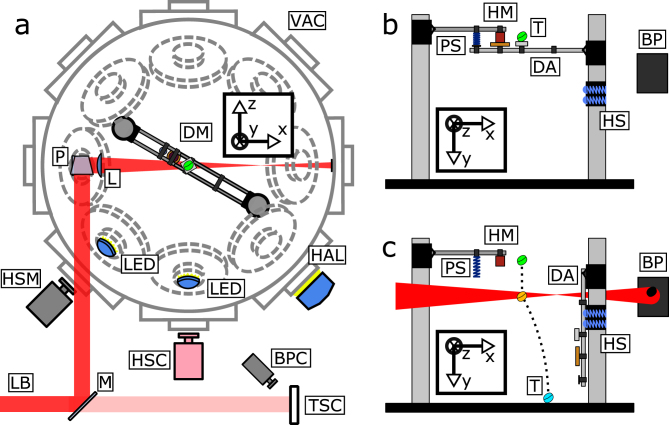
Sketch of the experimental setup. (**a**) Top view of the vacuum chamber. (**b**+**c**) View from color high-speed cameras perspective showing (**b**) the prepared dropping mechanism and (**c**) Laser irradiation with indicated falling path from green over orange to blue (along the dotted line). The global coordinate system is indicated by arrows entitled x,y and z. Abbreviations: VAC: vacuum chamber, DM: dropping mechanism, P: periscope, L: Lens, LED: LED-lamp, HAL: halogen lamp, HSM: high-speed camera monochrome, HSC: high-speed camera color, BPC: beam profiling camera, LB: Laser beam, M: mirror, TSC: PTFE screen, HM: holding magnet, T: target, BP: burn pattern foil, PS: pressure spring, DA: dropping arm, HS: holding springs.

In the experiment room (compare Fig. 5a) the laser beam is redirected to enter a vacuum chamber through an optical quality anti-reflective coated (AR) window from the top. The light within the chamber is, via a highly reflecting (HR) mirror, redirected to a horizontal propagation direction through the center of the chamber. Directly behind the mirror an AR coated three inch diameter lens (Thorlabs LA4246 with C-coating) with 500 mm focal length is used to reduce the beam diameter (64 mm) in the target plane to approximately 3 cm. The beam shape was verified by a burn pattern. The remaining laser beam is dumped on black anodized aluminum foil several ten centimeters after the intermediate focus. Beam polarization at the target was horizontal.

To keep track of the beam properties, for every single laser shot the time-dependent signal of the laser pulse was captured with a photodiode (Thorlabs DET01CFC). A beam centering analysis module gave information about the laser pulse energy prior to its amplification. The amplified beam was further analyzed by a PTFE scattering screen which again was exposed to leaking laser light at a folding mirror. The screen was monitored by a CMOS camera (Basler acA2000-50 gm) capturing information about the beam profile and relative intensity (Fig. 4b). In order to generate a reference to these relative intensities, four laser shots were documented dumping all pulse energy onto a calorimeter (Gentec QE95LP-H-MB-QED-D0). The average pulse energy was considered to equal the average pulse energy during the remaining experiments. These two-dimensional profiling measurements were accompanied by burn patterns. Typically these can be achieved by exposing thermal printing paper (Kodak Linagraph direct print type 1895) to the laser. A second type of burn pattern foil, black anodized aluminum foil, was used as a beam dump. After a full energy illumination it documented a shadowgraph of the free falling target at the moment of laser illumination (Fig. 4c).

In order to release the debris targets for free fall, a dropping arm was constructed (Fig. 5b,c) consisting mainly of Thorlabs standard opto-mechanical components. An anodized aluminum breadboard 15 cm × 60 cm in size was fixated within the vacuum chamber. On top of the breadboard, two opposing 1.5 inch diameter metal rods with an absolute height of 56 cm and 51 cm were mounted to hold the dropping mechanism. The lower rod held the dropping arm build with the optical rail system allowing for a flexible adjustment to the desired lengths. The arm was attached via two ball bearings (MDC Vacuum Limited - precision bearings) to grant its free movement away from the target. 10 cm below the arm a pair of tension springs was used to catch the arm at its lowest point and prevent it from being repelled towards the free falling target. The other rod held an electrically demagnetizing magnet and a pressure spring. In its start position the arm was held by the magnet, while bringing pressure onto the pressure spring. As soon as the magnet was demagnetized the pressure spring was able to push the arm downwards and accelerate at a much higher rate than the falling target was accelerated by gravity (compare Fig. 5b,c).

Target movement was captured by a pair of high-speed cameras. Both cameras were placed outside the vacuum chamber recording the free fall at 1000 fps and 0.5 ms exposure time through a glass window each. The color high-speed camera (MotionPro X3 - IDT, Inc.) was placed perpendicular to the laser beam path while the black and white high-speed camera (Redlake MotionScope M-3) was placed in between the incoming laser pulse and the the first camera at a 45° angle. The wide field objectives with f = 1.4 and 12 mm focal length (Thorlabs - MVL12M1) allowed to view the entire free fall path. Heat shielding glasses made from Shott KG5 with a diameter of 50 mm (Edmund Optics - 49-095) were attached to the objectives to shield the cameras from direct laser irradiation. Color sensitivity was not essential for the experiment.

Most targets were made of an aluminum alloy (AL6061) and differently shaped to investigate the influence of the targets shape on its impulse vector. Supplementary Table S2 summarizes the targets involved in the experiment. Surfaces were taken as received from the workshop for fine mechanics (compare Fig. 4a). Targets were cleaned using ethanol and handled with disposable gloves.

The tested aluminum plates were weighed before and after ablation. The difference in target mass *m*_target_ equals the mass lost to the ablation jet *m*_jet_. To generate a realistic estimate of the uncertainty in weighing, a blind test was performed by dropping three aluminum targets without laser irradiation in the evacuated chamber and measuring the introduced mass difference, which ideally should be expected to be zero. Due to time restrictions the blind test was performed with a delay of one day.

Synchronized triggering of all electrical systems as well as the basic clock signal of the NHELIX laser system was achieved with an Arduino Leonardo based self-designed trigger box. The Arduino Leonardo was extended by a custom shield providing driving ICs to drive several 50 Ohm terminated BNC lines and one 12 V electromagnet. The system was placed within a housing supporting 10 BNC connectors for final wiring. One extra output was foreseen to drive the electrically demagnetizing magnet. An external 12 V power supply was used to provide stand alone functionality. Furthermore, all lines except the electric magnet can be driven by the USB supply line, which was also used to document the trigger procedure via a serial interface protocol. The serial protocol allowed to securely switch between an armed and an unarmed status of the trigger system, without being sensitive to stray induced signal spikes. Once armed, the experimental process can be started by pushing a corresponding button on the outside of the trigger box.

After pushing the trigger button, the box sends a trigger signal to the NHELIX laser system, which starts a countdown to emit the laser pulse. At the same time the box starts counting down to the last 200 ms clock cycle before laser emission. After a delay of approximately 50 ms the electromagnet is demagnetized. As soon as the electrical contact between electromagnet and dropping arm seizes, the high-speed cameras are triggered and the system waits a predefined falling time of approximately 150 ms of free fall. Simultaneously the clock signal of the NHELIX system will be delayed by a few ms to fire the system at the specified time. Shortly before the laser event occurs, the photodiode and the beam profiling camera are triggered to capture the beam properties.

Camera calibration was introduced using Microsoft’s “Easycalib” tool based on the Zhang algorithm^40^ in combination with the preceding ImageJ Plugin “Optic Calib” by Peter Stierlen^41^. Tracking of markers was performed manually using ImageJ. Intermediate movements were interpolated and translated to time and position tables. These tables were evaluated by stereoscopic triangulation, determining the midpoint of the closest approach from corresponding projection rays. The algorithms were implemented in a custom Python script, which was followed by a script fitting the parameters of freedom for a rigid object: angular velocity ${\overrightarrow{\omega }}_{i}$ and velocity ${\overrightarrow{v}}_{i}$ before and after the laser irradiation. The used coordinate system is right handed as indicated in Fig. 5.

To give an additional estimate of the effects introduced by vacuum quality the aluminum plate experiments were performed at two different pressure levels. The higher level was chosen slightly below 2·10^−1^ mbar while the lower level was chosen slightly below 10^−4^ mbar.

### EXPEDIT

Our simulation tool EXPEDIT has been described earlier^28^. In short, the tool calculates momentum and angular momentum by discretizing both laser beam and debris surface into multiple rays and target surface elements, resp. Then, the imparted momenta can be computed by summation over the corresponding momentum elements that stem from the specific laser-matter interactions given by each ray and its corresponding surface element obtained by ray tracing. Hence, the local fluence at each point of the target surface can be taken into account for a precise calculation of laser-ablative momentum.

To compare the simulation to our experiments we added several interaction curves

1 $${c}_{m}({\rm{\Phi }})\approx \frac{{\rm{\Phi }}-{{\rm{\Phi }}}_{0}}{{\rm{\Delta }}{\rm{\Phi }}+({\rm{\Phi }}-{{\rm{\Phi }}}_{0})}\cdot b\cdot 12.46\cdot {A}^{\mathrm{7/16}}\cdot {(\frac{\sqrt{\tau }}{\lambda \cdot {\rm{\Phi }}})}^{c}$$

where *A* = 26.98 is the atomic mass of aluminum, *τ* the pulse length of the laser and Φ is the fluence, given in *J*/*cm*^2^. The parameters Φ_0_, b, c and ΔΦ were fitted to simulation results originating from the Polly-2T tool developed by M. Povarnitsyn at the Joint Institute of High Temperatures (JIHT) at the Russian Academy of Sciences (RAS), Moscow^31^. The expression in Eq. (1) is an approximated analytical function for momentum coupling taking into account a wide range of laser fluences (vaporization, transition and plasma regime) from the theory of laser-ablative momentum coupling depicted in great detail in a review about laser ablative propulsion^23^ (compare eq. (19)^23^ and eq. (39)^23^). Simulation results from Polly-2T are shown in Supplementary Fig. (S1) as symbols whereas line graphs indicate fitted curves with the parameters listed in Supplementary Table S3.

For the simulations *c*_*m*_(Φ) curves corresponding to a 0° angle of incidence were used as a standard. Tilted plates were simulated with the 30° curve and L-corners were simulated using the result for 45° angle of incidence.

The laser pulse energy and pulse duration deviated from shot to shot. Recording the leaking intensity profile of every laser pulse allowed to deduce direct pulse energy relations (Supplementary Table S1) and fluence distributions (compare Fig. 4b). Fitting a Gaussian standard distribution to the time profile of the photo diode additionally allowed to deduce the individual pulse duration *τ* (Supplementary Table S1) relevant for Equation 1.

In order to estimate the correct point of impact onto the target and by this reduce the error introduced from drop timing instabilities, we evaluated the tracked positions and orientations. The average position of the targets during irradiation was chosen as the center of the laser beam, which, as indicated by the burn patterns (compare Fig. 4c), is a valid assumption.

### Data availability

Further data generated during and/or analysed during the current study are available from the corresponding author on reasonable request.

## Electronic supplementary material

supplementary video 1

supplementary material
