# Supplementary material for: Experimental verification of high energy laser-generated impulse for remote laser control of space debris
Source: Sci Rep. 2018 May 31;8:8453. doi: 10.1038/s41598-018-26336-1 (PMC5981302; doi:10.1038/s41598-018-26336-1)
Supplement: Supplementary file 2 — supplementary material [file 41598_2018_26336_MOESM2_ESM.pdf]

Experimental verification of high energy laser-generated impulse for remote laser control of space debris

Raoul-Amadeus Lorbeer[1,*], Michael Zwilich[1], Miroslav Zabic[1], Stefan Scharring[1], Lukas Eisert[1], Jascha Wilken[1], Dennis Schumacher[2], Markus Roth[3], and Hans-Albert Eckel[1]

[1]German Aerospace Center - Deutsches Zentrum für Luft- und Raumfahrt e.V. (DLR), Institut für Technische Physik , Studien und Konzepte , Pfaffenwaldring 38-40 , 70569 Stuttgart, Germany
[2]GSI Helmholtzzentrum für Schwerionenforschung GmbH, Atomic, Plasma Physics and Application Planckstraße 1, 64291 Darmstadt, Germany
[3]Technische Universitt Darmstadt, Institut für Kernphysik Schlossgartenstraße 9, 64289 Darmstadt, Germany
[*]Raoul.Lorbeer@dlr.de

Supplementary Materials

Table S1, S2, S3
Fig S1
Movie S1

Supplementary tables

#	cd	m_f	Δm	τ	E_L	$\Delta\vec{v}$	$\Delta\vec{\omega}$
		[g]	[µg]	[ns]	[J]	[m/s]*	[2πrad$/s$] *
error		$\pm 10^{-4}$ g	$\pm$20µg	$\pm$0.15 ns	$\pm$3 J	$\pm$0.01 m/s*	$\pm\frac{2\pi}{4}$ rad$/s$ *
1	TL	3.0618	—	8.7	—	(0.31, -0.01, -0.15)	(0.03, 1.82, 0.87)
2	TL	3.0424	—	9.5	—	—	—
3	TL	3.0510	—	9.6	75.9	(0.32, -0.01, -0.05)	(-0.15, 0.40, 0.01)
4	TL	3.0410	—	10.4	80.6	—	—
5	AL	3.0239	—	11.7	81.5	(0.44, -0.04, -0.05)	(0.15, 0.73, 0.28)
6	AL	3.0951	—	11.3	81.3	(0.45, -0.04, -0.03)	(0.00, 0.76, 0.29)
7	AL	3.0715	—	10.9	62.6	(0.36, -0.02, -0.09)	(0.25, -0.53, -1.24)
8	AL	3.0150	—	11.1	90.1	(0.45, -0.04, 0.05)	(0.08, 2.52, 1.14)
9	CC	3.2598	—	11.2	82.8	(0.36, -0.02, -0.09)	(-0.16, 0.34, 0.35)
10	CC	3.2899	—	11.1	79.1	(0.36, -0.04, -0.05)	(0.02, 0.09, -0.29)
11	CC	3.4625	—	11.4	81.5	(0.34, -0.04, -0.05)	(0.21, -0.03, 0.16)
12	CC	3.4014	—	10.9	80.6	(0.35, -0.01, -0.07)	(-0.17, 0.19, 1.01)
13	TL	2.9759	—	9.7	86.2	(0.32, 0.00, -0.10)	(0.05, 0.92, 1.41)
14	SP	1.4700	—	9.4	89.7	—	—
15	P3	1.0895	-88	11.1	81.7	(0.86, 0.02, -0.75)	(2.93, 1.74, 4.01)
16	P3	1.1069	-72	10.8	81.0	(0.98, 0.09, -0.72)	(0.96, 1.31, 0.60)
17	P3	1.1005	-96	10.0	83.5	(1.00, 0.02, -0.71)	(0.85, 1.28, 1.17)
18	P3	1.0880	-84	10.4	83.6	(1.03, 0.07, -0.67)	(0.67, -0.22, 0.75)
19	P0	1.0871	-92	10.0	81.0	(1.45, 0.09, -0.07)	(-0.15, 6.01, 6.84)
20	P0	1.0684	-100	10.0	80.4	(1.33, 0.06, -0.09)	(-0.04, -0.35, 5.22)
21	P0	1.0871	-100	10.7	80.0	(1.29, 0.04, -0.12)	(-0.02, -0.30, 4.71)
22	P0	1.1014	-96	10.6	79.3	(1.25, 0.07, -0.07)	(-0.36, 5.14, 2.90)
23	PC	1.1154	—	10.4	92.8	(0.98, 0.01, -0.01)	(0.52, -1.70, 7.91)
24	AM	2.1346	—	9.6	83.0	(2.80, 0.08, 0.02)	(-0.95, -5.42, 19.47)
25	PS	3.0693	—	9.6	91.6	(0.51, 0.01, -0.04)	(-0.16, 0.68, 0.42)
26	DC	3.4014	—	11.8	86.6	(0.25, -0.02, 0.05)	(-0.24, 0.10, 0.17)
27	GB	2.1896	—	12.1	94.0	(-0.55, -0.05, -0.04)	(-0.61, 7.61, 2.00)

Table S1. Figures of merits for all 27 experiments. cd: experiment condition as defined in table S2; #: experiment no.; m_f : final material mass after ablation; Δm : mass difference after ablation; τ: pulse duration (full width half maximum); E_L: pulse energy; $\Delta\vec{v}$: change in velocity; $\Delta\vec{\omega}$: change in angular velocity; *shots #15 and #24 have errors below $5 \cdot 10^{-2}\, m/s$ and $2\pi\, rad/s$

target	abbreviation	material	orientation	# rep.	mass [g]
L-profile	TL	AL6061	bend to laser	5	3.036 ± 0.035
L-profile	AL	AL6061	bend from laser	4	3.051 ± 0.038
cylinder	CC	AL6061	centered	4	3.353 ± 0.095
cylinder	DC	AL6061	off-centered	1	3.401 ± 0.000
spring	SP	AL6061	centered	1	1.470 ± 0.000
plate	P0	AL6061	0° angle of incidence	5	1.086 ± 0.014
plate	P3	AL6061	30° angle of incidence	5	1.096 ± 0.009
plate	PC	copper	0° angle of incidence	1	1.115 ± 0.000
plate	PS	carbon steel	0° angle of incidence	1	3.069 ± 0.000
Arduino Mini	AM	PCB	0° angle of incidence off-centered	1	2.135 ± 0.000
gummibear green	GB	gelatin and sugar	centered	1	2.190 ± 0.000

Table S2. List of targets. Indicated are shape, material, orientation, number of repetitions and the average target mass after irradiation. Tolerances in mass are dominated by variations between single targets. Scale precision was in the range of several µg.

angle of incidence [deg]	$\Delta\Phi$ $[J/cm^2]$	b $[N/MW]$	c $[-]$	Φ_0 $[J/cm^2]$
0	4.740	0.192	0.593	3.029
15	3.671	0.191	0.551	3.000
30	1.962	0.189	0.468	2.974
45	0.992	0.182	0.414	2.970
60	1.236	0.165	0.424	2.102
75	1.297	0.174	0.235	1.498

Table S3. List of fit parameters to equation (**??**) reproducing the line graphs in fig. (S1).

Supplementary figures

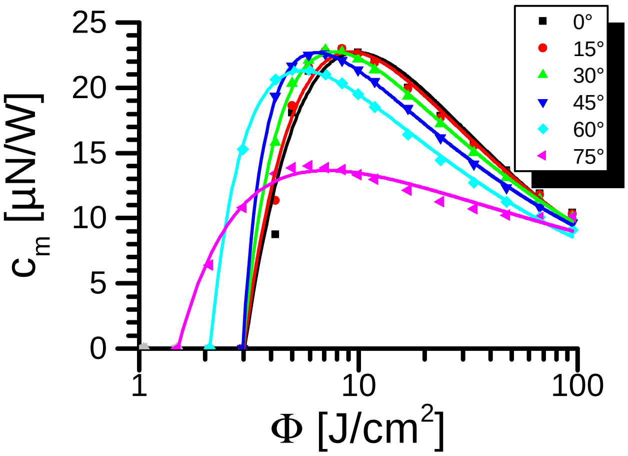

Figure S1. Impulse coupling coefficient $c_m(\Phi)$ over fluence Φ for several angles of incidence, p-polarized light at 1064 nm and 10 ns pulses. Symbols indicate simulation results for aluminum as target material with Polly-2T. Lines indicate fitted curves.

Supplementary movies

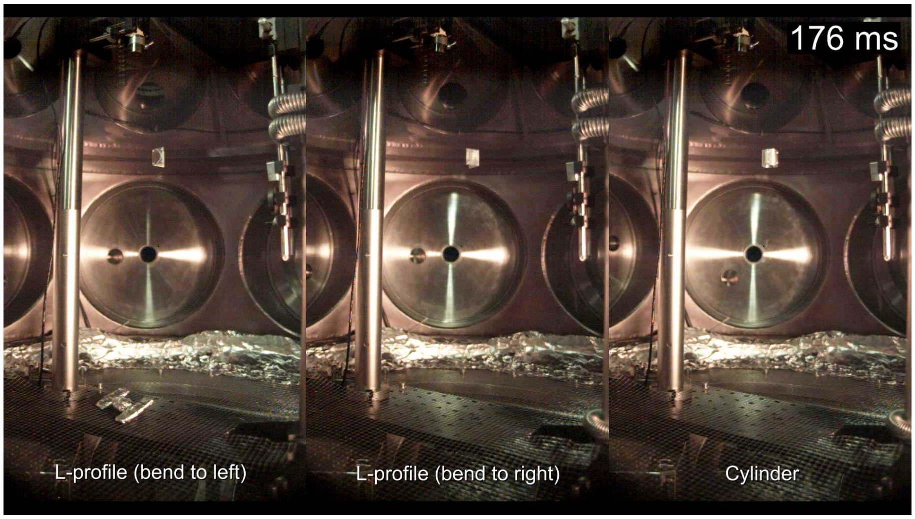

Movie S1. The supplementary movie shows slow motion videos of several targets. Shown are L-corner (left and right), Cylinder, aluminum-plate, copper-plate, Arduino mini, steel-plate, and gummy bear.
